# Living mulch enhances soil enzyme activities, nitrogen pools and water retention in giant reed (*Arundo donax* L.) plantations

**DOI:** 10.1038/s41598-024-51491-z

**Published:** 2024-01-19

**Authors:** Nevien Elhawat, Andrea Balla Kovács, Gabriella Antal, Erika Kurucz, Éva Domokos-Szabolcsy, Miklós Gábor Fári, Tarek Alshaal

**Affiliations:** 1 Department of Applied Plant Biology; Faculty of Agricultural and Food Sciences and Environmental Management, University of Debrecen, Böszörményi str. 138, 4032 Debrecen, Hungary; 2 Department of Biological and Environmental Sciences, Faculty of Home Economic, Al-Azhar University, Tanta, 31732 Egypt; 3 Institute of Agricultural Chemistry and Soil Science, FAFSEM, University of Debrecen, 4032 Debrecen, Hungary; 4 Institute of Horticulture University of Debrecen, 4032 Debrecen, Hungary; 5 Soil and Water Department, Faculty of Agriculture, University of Kafrelsheikh, Kafr El-Sheikh, 33516 Egypt

**Keywords:** Biodiversity, Plant sciences

## Abstract

Giant reed (*Arundo donax* L.) is one of the most well-studied perennial biomass crops because of its high productivity and potential to store carbon. Yet, little information on controlling weeds in giant reed plantations and their influences on the soil ecosystem is available. In the present study, three different weed control methods, i.e., intercropping (living mulch) with sweet clover (*Melilotus officinalis* L.), herbicide (glyphosate), and hoeing, were investigated in a 2-year giant reed farm. The intercropping presented significantly higher values (on average) of all the tested soil properties than herbicide and hoeing, except for the catalase activity and pH. The dehydrogenase, phosphatase, and urease activities in the soil under intercropping were higher than the herbicide by 75%, 65%, and 80% (on average), respectively. Also, the soil under intercropping had higher soil organic matter (SOM) and soil respiration than the herbicide by 20% and 25%, respectively. Intercropping also increased the content of N pools, i.e., NO_3_^−^˗N, NH_4_^+^˗N, Org-N, and Total-N by 517%, 356%, 38%, and 137%, respectively, compared to herbicide. These findings illustrated that controlling weeds in biomass plantations through legume intercropping brings benefits not only to soil properties but also to biomass productivity.

## Introduction

Day after day, the interest in biomass crops as an alternative to fossil fuels or at least to share a significant portion of the world energy portfolio considerably increases, particularly after the global energy crisis and amplified energy prices^1,2^. Nevertheless, biomass production should be accompanied by low agronomic inputs, such as fertilizers and agrochemicals (e.g., pesticides and herbicides), to support the energy balance and make it positive^3^. Controlling weeds is of importance since weeds can bring pathogens to the growing crop or act as a host of damaging pests and insects, as well as to avoid competition with the main crop for nutrients, light, and water and thus reduce yield and its quality^4^. Also, controlling weeds ensures low competition for nutrients and water^5^. Although biomass crops are characterized by rapid growth rates, the first year of cultivation is sensitive as it is the determining factor for the establishment of a successful farm with a targeted plant density and vigorous plant growth^6^.

Controlling weeds substantially alters soil properties, including the structure and functions of soil microorganisms^7^. For instance, 30% more operational taxonomic units were reported in untapped soil compared to cultivated soil^8^. Likewise, 1000 microbial species were measured per one gram of uncultivated soil, while cultivated soil showed 140–150 species per gram of soil^9^. The intensive soil disturbance through tillage and/ or hoeing accelerates soil organic matter (SOM) loss due to the fast mineralization process^10^. Moreover, the extensive and indiscriminate use of herbicides has adverse consequences on the structure of soil biota, soil functionality, ecosystem sustainability, human and animal health, and food safety and security. For instance, the World Health Organization (WHO) reported more than three million cases of agrochemical poisoning in developing countries^11^.

Several indicators have been proposed as potential tools to assess changes in soil health due to applied management practices; however, soil biochemical traits are the most sensitive signs^12^. Soil enzymes catalyze several biochemical processes such as the decomposition of plant residues, nitrogen fixation, nitrification, denitrification, transformation of different organic compounds, nutrient cycling, and removing toxic compounds. Therefore, measuring the activity of soil enzymes, more often than not, is a very indicative tool for soil health and agronomic management^13^.

Dehydrogenase activity is used as a function of the active soil microorganisms since it is only active in living cells. Furthermore, its activity is strongly related to the composition and activity of soil microorganisms, SOM, and carbon cycling^14^. Therefore, dehydrogenase activity has been widely used as an indicator of soil health and the efficiency of agronomic practices^15^. Soil disturbance negatively affects dehydrogenase activity. Direct seed sowing resulted in higher activity of dehydrogenase than the conventional cultivation method that is based on the moldboard plow (0 to 20 cm) using a traditional soil tillage tool^16^. Furthermore, soils with minimum or no tillage displayed higher dehydrogenase activity than conventional tillage^17^. Phosphatase is responsible for releasing phosphorus (P) from its organic compounds, making them available for plant uptake. Living organisms in the soil, such as plant roots, bacteria, and fungi, are the only sources of soil phosphatase. Therefore, high phosphatase activity corresponds to soil health and the phytoavailability of P^18^. Soil with no tillage exhibited higher phosphatase activity than conventional and minimum tillage soils^19^. Urease activity depends heavily on the way the soil is cultivated. It shares a significant portion in nitrogen (N) cycling^20^. Soil managed at minimum tillage showed twofold higher urease activity than conventional agriculture^13^. Likewise, soil with no tillage revealed higher urease activity than conventional and minimum tillage soils^19^. However, soil enzyme activity considerably depends on the degree of soil disturbance that increases the oxidation of organic matter.

The content of SOM is a very useful tool for measuring soil fertility and health. The main portion of SOM comes from plant residues, while soil microbial biomass carbon and nitrogen represent a small portion of SOM^13^. Organic manures represent a substantial exogenous source of SOM, besides their benefits in improving soil microbial biomass and enzyme activities^21^. Moreover, Zhang et al.^22^ studied the effect of ryegrass and straw mulch in an apple orchard on the content of SOM; they reported that straw mulch increased more the SOM content compared to the ryegrass mulch system.

Intercropping, also known as living mulch, is commonly used in various cultivation systems to provide a range of benefits to the soil and crops. The use of intercropping is prevalent in agroforestry systems, orchards and vineyards, fruit and berry crops, and vegetable. Living mulch, particularly using legumes such as clover and vetch, can have several positive effects on soil ecosystem, including soil erosion control, organic matter addition, weed suppression, water conservation, temperature regulation, microbial activity, and biodiversity. However, there is limited information on how the soil ecosystem and its functionality respond to different weed managements in giant reed (*Arundo donax* L.) plantations. In the present study, we hypothesized that intercropping (using sweet clover) in one-year-old giant reed plantation would efficiently suppress weeds, considerably improve soil fertility through symbiotic N_2_ fixation, and ultimately enhance growth and yield of giant reed plants compared to conventional practices, i.e., hoeing and herbicides. Changes in soil physical, chemical, and biochemical properties, after controlling weeds by intercropping, hoeing, or herbicide was evaluated. The activity of soil enzymes and soil N pools were among the main tested parameters.

## Results

### Biochemical soil properties under different weed control methods

#### Soil enzyme activities

The activity of soil enzymes, i.e., dehydrogenase (μg TPF g soil^−1^ h^−1^), urease (mg NH_4_^+^ 100 g soil^−1^ 24 h^−1^), phosphatase (μg PNP g soil^−1^ h^−1^), and catalase (mL O_2_ 2 min^−1^), significantly varied according to the sampling time and applied weed control technique (Table 1). Dehydrogenase and urease enzymes displayed significantly higher activities (255 and 906, respectively) in autumn than in spring (115 and 98, respectively). On the contrary, phosphatase and catalase enzymes showed their highest activities in spring (66.7 and 40.8, respectively). Controlling weeds in giant reed plantations by sweet clover intercropping technique significantly induced the activity of dehydrogenase, urease, and phosphatase enzymes compared to herbicide and hoeing treatments. Moreover, the differences between herbicide and hoeing treatments were not significant. The activities of dehydrogenase, urease, and phosphatase were 263, 738, and 79.0, respectively, under the intercropping treatment, while they were 142, 358, and 44.2, respectively, under the hoeing treatment. Catalase enzyme showed a different response to the weed-controlling methods compared to the other soil enzymes, where the differences in catalase activity were insignificant. Concerning the interaction between sampling time and weed control technique, intercropping treatment during autumn time showed the significantly highest activities of dehydrogenase (375) and urease (1340), while the same treatment displayed the highest activity of phosphatase (93.3) in spring. Hoeing and herbicide treatments exhibited lower soil enzyme activities than intercropping treatment in both sampling times.

**Table 1:** Seasonal variation of soil enzyme activity in a 2-year giant reed (*Arundo donax* L.) plantations under different weed control managements, i.e., sweet clover intercropping, herbicide, and hoeing.

	Dehydrogenase (μg TPF g soil^−1^ h^−1^)	Urease (mg NH_4_^+^ 100 g soil^−1^ 24 h^−1^)	Phosphatase (μg PNP g soil^−1^ h^−1^)	Catalase (mL O_2_ 2 min^−1^)
Season (sampling time)				
Spring	115 ± 13 b	98 ± 11 b	66.7 ± 3.93 a	40.8 ± 2.11 a
Autumn	255 ± 21 a	906 ± 33 a	47.4 ± 2.51 b	8.0 ± 0.07 b
Weed control method				
Intercropping	263 ± 11 a	738 ± 32 a	79.0 ± 3.61 a	20.4 ± 0.92 a
Herbicide	150 ± 16 b	409 ± 19 b	48.0 ± 3.50 b	24.4 ± 1.23 a
Hoeing	142 ± 13 b	358 ± 14 b	44.2 ± 2.55 b	28.3 ± 1.11 a
Interaction				
Spring				
Intercropping	151 ± 1 bc	137 ± 50 c	93.3 ± 4.67 a	33.5 ± 5.05 ab
Herbicide	110 ± 3c	81 ± 30 c	58.8 ± 4.52 b	40.1 ± 5.80 a
Hoeing	83 ± 5 c	77 ± 00 c	48.1 ± 2.60 c	48.6 ± 7.10 a
Autumn				
Intercropping	375 ± 55 a	1340 ± 44 a	64.7 ± 2.55 b	7.4 ± 1.77 b
Herbicide	190 ± 25 b	737 ± 91 b	37.2 ± 2.48 d	8.7 ± 0.65 b
Hoeing	200 ± 14 b	641 ± 56 b	40.3 ± 2.49 cd	8.0 ± 1.14 b
Two-way ANOVA				
Season	***	***	***	***
Weed control	***	***	***	ns
Interaction	***	***	***	ns

Means in the same column followed by the different letters are significant according to Tukey’s test (*P* ≤ *0.05*). Data are mean ± SD and n = 9. *** denotes significance at *P* < 0.001; ns denotes insignificant at *P* ≤ 0.05.

#### Soil organic matter and soil respiration

**Figure 1:**
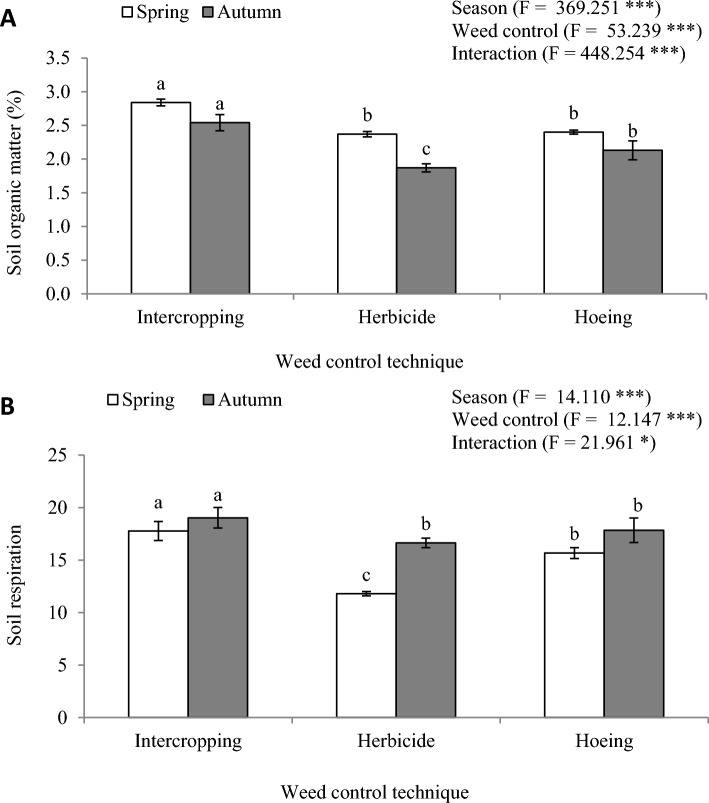
(**A**) soil organic matter (%) and (**B**) soil respiration (mg CO_2_ 100g soil^−1^ 10 days^−1^) in a 2-year giant reed (*Arundo donax* L.) plantations under different managements of weed control, i.e., sweet clover intercropping, herbicide, and hoeing. Different letters on bars of the same season are significant according to Tukey’s test (P ≤ 0.05). Data are mean ± SD and n = 9. *** and * denote significance at P ≤ 0.001 and P ≤ 0.05, respectively.

Statistical analyses revealed that the content of soil organic matter (SOM; %) significantly varied according to the sampling time (Fig. 1A). The SOM content in spring (2.54) was significantly higher than those measured in autumn (2.18). On the other hand, controlling weeds in the giant reed plantations by intercropping technique showed significantly higher SOM content (2.69) than herbicide (2.12) and hoeing (2.27) methods. Also, the differences in the SOM content of herbicide and hoeing treatments were insignificant. The results of the interaction between sampling times and weed control methods showed that the intercropping method in spring resulted in the highest SOM content (2.84), while the application of herbicide to control weeds in autumn the lowest SOM content (1.87).

The production of CO_2_—as an indicator of soil respiration—refers to the activity of soil organisms. Sampling time was a significant factor affecting the soil respiration (mg CO_2_ 100g soil^−1^ 10 days^−1^), where soil respiration in autumn was significantly higher (17.84) than measured in spring (15.08). Furthermore, soil respiration showed significant dependence on chosen technique for controlling weeds in the giant reed plantations. The utilization of sweet clover as an intercropping plant to control weeds significantly resulted in a higher soil respiration rate (18.40) than hoeing (16.76) and herbicide (14.22). The intercropping treatment in autumn was the best among all the weed control treatments in the spring and autumn seasons, recording a soil respiration rate of 19.03. The lowest soil respiration rate was 11.80 and corresponded to herbicide treatment in spring. The application of herbicide to control weeds had the worst impact on the activity of soil organisms as it recorded the lowest rate of soil respiration in both sampling times (Fig. 1B).

### Nutritional status of soil under different weed control methods

#### Nitrogen pools

Dynamics of different nitrogen (N) forms, i.e., nitrate (NO_3_^−^-N), ammonium (NH_4_^+^-N), organic nitrogen (Org-N), and total nitrogen (total-N), varied significantly according to the applied weed control method and to a lesser extent to sampling time (Table 2). All determined N pools (mg kg^−1^ soil) displayed significantly higher contents in spring. The contents of NO_3_^−^-N, NH_4_^+^-N, org-N, and total-N in spring were 9.03, 2.62, 3.54, and 15.2, respectively, whereas, in autumn, they were 8.84, 2.37, 3.43, and 14.7, respectively. The employment of a legume crop (sweet clover) in an intercropping system for controlling weeds in giant reed plantations significantly enhanced the dynamics of different soil N forms. The intercropping method resulted in the highest contents of NO_3_^−^-N, NH_4_^+^-N, org-N, and total-N, which are 17.03, 4.19, 4.57, and 25.8, respectively. The herbicide treatment exhibited a significantly higher content of N forms than the hoeing treatment, except for the NH_4_^+^-N form, where both treatments resulted in almost the same value.

**Table 2:** Seasonal variation of soil nitrogen pools (mg kg^−1^) in a 2-year giant reed (*Arundo donax* L.) plantations under different weed control managements, i.e., sweet clover intercropping, herbicide, and hoeing.

	NO_3_^−^˗N	NH_4_^+^˗N	Organic˗N	Total˗N
Season (sampling time)				
Spring	9.03 ± 0.02 a	2.62 ± 0.03 a	3.54 ± 0.08 a	15.2 ± 0.06 a
Autumn	8.84 ± 0.01 b	2.37 ± 0.01 b	3.43 ± 0.06 b	14.7 ± 0.05 b
Weed control method				
Intercropping	17.03 ± 0.06 a	4.19 ± 0.02 a	4.57 ± 0.02 a	25.8 ± 0.02 a
Herbicide	5.93 ± 0.02 b	1.64 ± 0.01 b	3.30 ± 0.04 b	10.9 ± 0.03 b
Hoeing	3.86 ± 0.04 c	1.66 ± 0.00 b	2.60 ± 0.03 c	8.1 ± 0.03 b
Interaction				
Spring				
Intercropping	17.23 ± 0.07 a	4.34 ± 0.03 a	4.67 ± 0.05 a	26.2 ± 0.04 a
Herbicide	5.98 ± 0.08 c	1.71 ± 0.05 c	3.35 ± 0.06 c	11.0 ± 0.06 c
Hoeing	3.89 ± 0.09 d	1.81 ± 0.05 c	2.62 ± 0.09 d	8.3 ± 0.06 e
Autumn				
Intercropping	16.83 ± 0.06 b	4.04 ± 0.01 b	4.47 ± 0.07 b	25.3 ± 0.13 b
Herbicide	5.88 ± 0.11 c	1.57 ± 0.03 d	3.25 ± 0.05 c	10.7 ± 0.03 d
Hoeing	3.82 ± 0.07 d	1.51 ± 0.03 d	2.57 ± 0.05 d	7.9 ± 0.01 f.
Two-way ANOVA				
Season	***	***	**	***
Weed control	***	***	***	***
Interaction	***	***	**	***

Means in the same column followed by the different letters are significant according to Tukey’s test (*P* ≤ 0.05). Data are mean ± SD and n = 9. *** and ** denote significance at *P* ≤ 0.001 and P ≤ 0.01, respectively.

Concerning the interaction between sampling times and weed control methods, the intercropping treatment revealed significantly higher contents of different N forms than herbicide and hoeing treatments across both seasons (i.e., spring and autumn). Also, the herbicide treatment displayed significantly higher values of N forms than the hoeing treatment, except for the NH_4_^+^-N form. The effect of intercropping treatment on N forms compared to herbicide and hoeing treatments was almost the same in spring and autumn. The intercropping treatment increased the contents of NO_3_^−^-N, NH_4_^+^-N, org-N, and total-N by almost 3-, 2.5-, 1.5-, and 2.5-fold, respectively, than herbicide treatment and by almost 4.5-, 2.5-, 2-, and threefold, respectively, than hoeing treatment, regardless of the sampling time.

#### Alterations in extractable soil P and K contents

Statistical analyses revealed that the contents of soil extractable P (mg 100 g^−1^ soil) and available K (mg kg^−1^) depended significantly on both sampling time and weed control method (Table 3). Overall, the extractable P content in autumn (74.6) was significantly higher than in spring (60.7). The intercropping technique resulted in the highest extractable P content (69.6), followed by hoeing (67.9) and herbicide methods (65.3). During spring, the hoeing method showed the highest extractable P content (72.1). Although the herbicide method exhibited the highest extractable P content in autumn (88.0), it revealed the lowest extractable P content in spring (42.7). However, the seasonal variations in the extractable P content under intercropping and hoeing methods were slighter than in the case of the herbicide method.

**Table 3:** Seasonal variations of some soil physicochemical properties in a 2-year giant reed (*Arundo donax* L.) plantations under different weed control managements, i.e., sweet clover intercropping, herbicide, and hoeing.

	Saturation %	Electrical conductivity (dS m^−1^)	pH	Extractable P (mg 100 g^−1^ soil)	Available K (mg kg^−1^ soil)
Season (sampling time)					
Spring	39.0 ± 0.36 a	0.527 ± 0.007 b	7.41 ± 0.01 a	60.7 ± 1.51 b	598 ± 2.9 a
Autumn	38.0 ± 0.41 b	0.543 ± 0.004 a	7.14 ± 0.01 b	74.6 ± 1.92 a	399 ± 1.9 b
Weed control method					
Intercropping	41.3 ± 0.34 a	0.617 ± 0.006 a	7.15 ± 0.02 b	69.6 ± 1.12 a	581 ± 2.2 a
Herbicide	37.0 ± 0.29 b	0.500 ± 0.005 b	7.32 ± 0.00 a	65.3 ± 0.96 c	463 ± 5.2 b
Hoeing	37.3 ± 0.31 b	0.488 ± 0.008 c	7.71 ± 0.01 a	67.9 ± 1.04 b	451 ± 4.3 b
Interaction					
Spring					
Intercropping	42.0 ± 0.40 a A	0.593 ± 0.006 a B	7.23 ± 0.02 c B	67.2 ± 1.00 b BC	675 ± 12.3 a A
Herbicide	37.0 ± 0.29 b CD	0.486 ± 0.012 b DE	7.46 ± 0.02 b A	42.7 ± 0.95 c D	492 ± 12.2 c C
Hoeing	38.0 ± 0.55 b C	0.502 ± 0.003 b CD	7.54 ± 0.00 a A	72.1 ± 0.50 a B	627 ± 11.7 b B
Autumn					
Intercropping	40.6 ± 0.21 a B	0.640 ± 0.008 a A	7.06 ± 0.05 a D	72.1 ± 0.70 b B	487 ± 13.1 a C
Herbicide	37.0 ± 0.40 b CD	0.513 ± 0.005 b C	7.17 ± 0.08 a C	88.0 ± 1.91 a A	434 ± 0.0 b D
Hoeing	36.5 ± 0.53 b D	0.475 ± 0.005 c E	7.19 ± 0.04 a C	63.6 ± 1.38 c C	275 ± 0.0 c E
Two-way ANOVA					
Season	***	***	***	***	***
Weed control	***	***	***	***	***
Interaction	***	***	**	***	***

Means in the same column followed by the different letters are significant according to Tukey’s test (*P* ≤ 0.05). Data are mean ± SD and n = 9. *** and ** denote significance at *P* ≤ 0.001 and P ≤ 0.01, respectively.

Soil available K content was significantly higher in spring (598) than in autumn (399). Moreover, the intercropping method resulted in the highest content of available K (581) compared to the hoeing (463) and herbicide (451) methods, regardless of the sampling time. The differences between herbicide and hoeing methods were not significant. The highest available K content across the spring and autumn seasons corresponded to the intercropping treatment, recording 675 and 487, respectively. The hoeing method displayed the second-highest available K content in spring (627), while the herbicide method showed the second-highest available K content in autumn (434).

### Variations in some chemical properties of soil under different weed control methods

#### *Soil electrical conductivity* (EC_e_)

The EC_e_ (dS m^−1^) of soil solution varied significantly according to the sampling time, where it was 0.527 (in spring) and 0.543 (in autumn). Also, the method applied to control weeds in the giant reed plantations significantly influenced the EC_e_, where the intercropping method displayed the highest EC_e_ value (0.617), while the lowest measured EC_e_ (0.488) corresponded to the hoeing method (Table 3). The highest EC_e_ values in spring (0.593) and autumn (0.640) were measured after controlling weeds by the intercropping method. Both herbicide and hoeing methods displayed a similar EC_e_ during spring, whereas, in autumn, the hoeing method exhibited a lower EC_e_ (0.475) than the herbicide method (0.513).

#### Soil pH

Soil pH measured in spring (7.41) time was significantly higher than those measured in autumn (7.14), regardless of the weed control method. Overall, controlling weeds in the giant reed plantations by the intercropping method resulted in the lowest measured soil pH (7.15), while the hoeing method revealed a soil pH of 7.71 as the highest measured soil pH. Applying herbicide for weed control resulted in a soil pH of 7.32 (Table 3). Within spring, the hoeing method showed the highest soil pH (7.54), whereas the intercropping method exhibited the lowest soil pH (7.23). Likewise, in autumn, the highest soil pH (7.19) corresponded to the hoeing method, while the intercropping method resulted in the lowest soil pH (7.06). Statistical analyses illustrated significant differences among weed control methods and sampling times.

### Soil water retention capacity under different weed control methods

#### Soil saturation percentage

The soil saturation percentage (%) significantly varied according to the method applied to control weeds in the giant reed plantation (Table 3). Sampling time also significantly impacted the saturation %, where, in spring, the saturation % was significantly higher (39.0%) than in autumn (38.0%). While the hoeing and herbicide methods displayed almost the same soil saturation of ~ 37.0%, the intercropping technique resulted in the highest saturation percentage of 41.3%, regardless of the sampling time. Moreover, the intercropping method resulted in the significantly highest saturation % across the spring and autumn seasons, recording 42.0% and 40.6%, respectively. The herbicide method exhibited the lowest soil saturation % (37.0) in spring, while the hoeing method displayed the lowest soil saturation method (36.5) during autumn. However, the differences between herbicide and hoeing methods across the spring and autumn seasons were statistically insignificant.

## Discussion

Controlling weeds represents a cornerstone constituent of total agronomic management; with other activities like pest and disease control, fertilization, and irrigation, it guarantees the success of farm establishment and crop production. In the present study, the evaluated weed control methods, i.e., sweet clover intercropping, herbicide, and hoeing, successfully controlled weeds in the giant reed plantations. However, soil properties showed different responses to the tested weed control methods. Also, the time of soil sampling significantly influenced the soil traits.

Plant roots, plant residues, soil microorganisms, and soil animals are sources of soil enzymes, which form complexes with organic matter, clay, or clay-humus complex for stabilization^18^. Thus, SOM and soil texture are among the main controllers of soil enzyme activities. Over the two sampling times, sweet clover intercropping showed superiority to herbicide and hoeing concerning the activity of soil enzymes, where intercropping significantly increased the dehydrogenase, urease, and phosphatase activities.

The beneficial effects of sweet clover intercropping treatment on soil enzyme activities could be ascribed to root exudates, nitrogen fixation, and organic carbon^23^ that are added into the bulk soil around giant reed plants by sweet clover plants. In our experiment, SOM under sweet clover intercropping was the highest in spring and autumn samples (2.84% and 2.54%, respectively) compared to herbicide and hoeing. Similarly, Qian et al.^23^ stated similar positive effects of using white clover as a living mulch candidate on soil enzyme activities, recording higher activities of soil invertase, urease, and alkaline phosphatase.

Legume green manures have several advantages over non-legume green manures, including symbiotic N_2_ fixation, improved soil aggregation, enhanced soil water retention, controlled disease, and CO_2_ sequestration^24^. In the present study, sweet clover was applied primarily to control weeds in the giant reed plantations; yet, it served as potential green manure due to its high plant residues incorporated into the soil as indicated by high SOM contents (Fig. 1A). Zhang et al.^25^, in their 20-year fertilization experiment under greenhouse conditions, reported that the single application of horse manure compost (75 ton ha^−1^) and combined application of horse manure compost with N added as urea (either 300 or 600 kg N ha^−1^) significantly increased the activity of soil enzymes, SOM, and microbial biomass C and N compared to unfertilized control. They also documented lower soil enzyme activities in the case of the single application of inorganic N fertilizers, regardless of its application rate. The main driving factor for such findings is the SOM, which was lower in plots fertilized with synthetic N fertilizers due to the low incorporated plant residues into soil compared to those that received horse manure compost.

Soil enzyme activities depend on many factors, including plant species, soil moisture content, soil temperature, pH, SOM content, and salinity^26^. In the present study, controlling weeds through sweet clover intercropping in the giant reed field enhanced the physicochemical properties of soil as it increased the content of soil moisture, SOM, nitrogen forms, and potassium; while it reduced the soil pH. These changes create better growth conditions for soil microbes and consequently improve their functions, such as the activity of soil enzymes.

On the other hand, soil disturbance and herbicide application negatively influence soil enzyme activities. In our study, suppressing weeds by either herbicide or hoeing significantly declined activities of dehydrogenase, phosphatase, and urease at both sampling times in comparison with sweet clover intercropping; yet, a reversal of the effect on catalase activity was observed. Nguyen et al.^27^ cited a higher reduction in some soil enzyme activities after treating the soil with glyphosate herbicide than in the case of Roundup CT^®^ (a glyphosate formulation), particularly after three days of the application.

The mechanism of glyphosate depends on the inhibition of 5-enolpyruvylshikimate-3-phosphate synthase (EPSPS), a cornerstone enzyme in the shikimate pathway involved in aromatic amino acids synthesis in plants and several living organisms^28^. Lots of soil microorganisms also use the shikimic acid pathway for the biosynthesis of folic acid and aromatic amino acids (i.e., tryptophan, tyrosine, and phenylalanine); therefore, the application of herbicide glyphosate would be more likely to reduce microbial proliferation and diminish their functions including soil enzymes^27^. However, the impact of glyphosate on soil microflora in published studies varies from positive to neutral or negative.

Soil microorganisms of the surface horizon (0–10 cm) are the most active^29^; therefore, disturbing this layer would significantly affect the activity of soil microflora. For instance, soil tillage significantly lowered the activity of protease, arylsulfatase, cellulase, and phenoloxidase enzymes in the 0–10 cm soil layer of New Jersey Pinelands^30^. Likewise, conventional tillage of soil with maize-wheat rotation significantly caused a reduction of 40, 51, 74, 31, and 39% in activities of amylase, cellulase, arylsulfatase, acid phosphatase, and alkaline phosphatase, respectively, in the soil surface horizon (0–5 cm) compared to a zero tillage system^15^. De Barros et al.^31^ cited that changing the land use of forest into pasture combined with intensive anthropogenic disturbance significantly decreased the activity of urease, and alkaline phosphatase by 66 and 62%, respectively. The structure and functions of soil microorganisms heavily rely on the SOM, which mainly accumulates in the upper soil horizon (0–5 cm). Consequently, disturbance of this layer would be more likely to redistribute the SOM in the soil profile and accelerate its loss^10^, leading to a substantial reduction in the activities of soil microbes.

The emitted CO_2_ when soil microorganisms and plant roots respire is referred to as soil respiration. However, it is an effective measure of the overall activity of soil microbes and the breaking down of organic substances^32^. In the present study, controlling weeds with herbicide considerably diminished soil respiration in both sampling times compared to the other treatments, although it was applied once. Otherwise, the enhancement in soil respiration by sweet clover intercropping treatment is likely to occur because of the root exudates, plant residues, increased soil moisture, and soil nutrient levels, which induce the proliferation of soil microbes and improve their bioprocesses. Consistent with our data, Técher et al.^33^ documented higher rates of soil respiration in rhizosphere soil compared to bulk soil, which is less affected by root exudates. Xi et al.^34^ reported higher metabolic activity of carbon in orchard soil covered with living mulch; they ascribed this induced effect to the increased number of soil microbes due to living mulch. Likewise, white clover intercropping significantly enhanced soil respiration in maize soil^35^.

The SOM is one of the main controlling factors of soil fertility and plant nutrition. Indeed, SOM variation can be directly related to root exudation, plant residues, and microbial activities, which significantly affect the availability of soil nutrients. The intensive soil disturbance through plowing or hoeing accelerates SOM loss due to the fast mineralization process^10^. Intensive monoculture, soil tillage, and removing plant residues all cause a reduction in SOM content^36^. No significant differences were recorded in the SOM content of herbicide and hoeing treatments, probably because of the lower microbial activity due to the herbicide application and higher oxidation rates of organic matter due to hoeing. In this case, direct input of organic substances via sweet clover intercropping enhances the soil microbial activity, increases SOM content, promotes the nutrient cycle, and induces plant growth^37^. Meena et al.^38^ cited a reduction in SOM content in conventional maize- chickpea system compared to zero tillage due to the disturbance of the surface layer (0–15 cm) of the soil profile, where organic substances/ plant residues accumulate. On the other hand, soil microorganisms are responsible for the transformation and mineralization of the plant residues; therefore, the reduction in the numbers of soil microbes and their activities leads to lower SOM content^32^.

Recently, the interest in farming biomass crops has increased to avoid competition with traditional strategic crops^5^. Also, using plant species with low input requirements represents an additional advantage to the biomass production approach. Therefore, indigenous soil fertility and nutrient availability are growth-determining factors, particularly in the first year of establishment of a biomass farm, to ensure better plant growth and reduce the period required to reach the highest productivity^2^. Nitrogen is the main limiting nutritional element of plant growth, and its content is strongly associated with SOM content^23^. In the present study, an overall positive effect of sweet clover intercropping treatment on soil properties, i.e., soil enzyme activities, pH, soil moisture, and SOM content, was recorded in spring and autumn soil samples.

Moreover, weed control through intercropping management using sweet clover could have several positive consequences on the growth and development of giant reed plants. Besides the successful weed control, intercropping adds substantial amounts of atmospheric N_2_ to the soil through the symbiotic N_2_ fixation process. Hence, higher rates of mineralization and nitrification processes are expected due to the enhancement in the activity of soil microbes resulting in high content of NH_4_^+^ and NO_3_^−^. This finding is supported by high activities of dehydrogenase, phosphatase, and urease under sweet clover intercropping treatment compared to herbicide and/ or hoeing treatments (Table 1). An increase of 16–44% in SOM content and 50% in total N content due to covering the soil with living mulch using white clover was cited by Qian et al.^23^. Fiorentino et al.^37^ reported an abundance of the amoA gene in soil samples collected from giant reed plantations, indicating the promoting effect of giant reed on ammonia-oxidizing bacteria. They also measured plenty of the nifH genes in brownfield soil cultivated with giant reed plants. In the current study, the sweet clover intercropping exhibited the highest contents of different N forms. This result was in line with the SOM content, demonstrating that C and N cycling bacteria can be used to detect the impact of the different ways of weed control soils cultivated with bioenergy plants^39^.

In the present study, sampling time largely influenced soil pH, while to a lesser extent, the weed control method affected soil pH. The pH value was significantly higher in spring samples than in autumn. However, sweet clover intercropping treatment recorded lower pH values than glyphosate herbicide and hoeing treatments, regardless of the sampling times. Similar results were cited by Omer et al.^40^, who documented that pH was higher in winter and spring than in autumn. They also mentioned that the soil pH depended on plant species; for example, alfalfa (*Medicago sativa* L.) recorded lower pH than cotton (*Gossypium hirsutum*). Soil pH fluctuates according to soil moisture content^41^, where it usually increases to its highest value in midwinter and then gradually decreases, recording its lowest value in summer as the soil becomes drier^42^. In our experiment, the soil moisture content was higher in spring than in autumn; thus, higher pH values were noticed in spring. Also, K^+^ (alkali metal ion) content was higher in spring than in autumn, leading to a higher pH value.

Soil moisture content is one of the determining factors of EC_e_; however, plant roots cannot be ignored as an additional EC_e_-controlling factor. Rao et al.^43^ stated that the EC_e_ of soil replete with plant roots is slightly different from those with less plant root density. In our present study, both herbicide and hoeing treatments showed similar EC_e_ values, but lower than that corresponded to sweet clover intercropping treatment, which is rich in a fibrous root system. The high EC_e_ value, due to sweet clover intercropping, may be attributed to the metabolites secreted from the root system of sweet clover plants. However, EC_e_ displayed a hesitant response to the soil moisture content and sampling time.

Based on the discussed results above, it could be recommended that in the early stage of giant reed plantation, controlling weeds through living mulch intercropping using legumes, i.e., sweet clover, will have many positive and desired effects not only on soil properties but also on biomass productivity. The enhancement in soil properties, i.e., pH, water retention, enzyme activities, SOM content, and nutritional status, surely leads to better plant growth and higher biomass yield.

## Methods

### Source of plant materials

The plant material used in the current study was somatic embryo-derived plantlets of the Blossom ecotype of giant reed (*Arundo donax* L.) obtained from the University of South Carolina^44,45^ and propagated in the Ottó Orsós Laboratory, Department of Plant Biotechnology, Debrecen University, Hungary. Sterile plantlets were acclimatized first under greenhouse conditions and then transplanted in the open field.

### Experimental location and description

A field experiment was established in July 2013 at the demonstration Garden of Debrecen University, Debrecen, Hungary (47° 32′ 0″ N; 21° 38′ 0″ E). The experiment was arranged in a Randomized Complete Block design with three replicates making a total of 9 experimental plots. The plot area was 28 m^2^ consisting of four rows with one meter between rows and one meter between plants within a row. The experimental soil is a typical Chernozem soil with the following physicochemical properties: pH 7.63 ± 0.05; EC_e_ 0.45 ± 0.02 dS m^−1^; SOM 1.7 ± 0.11%; NH_4_^+^-N 1.8 ± 0.02 mg kg^−1^; extractable P 36.3 ± 1.88 mg kg^−1^; available K 237 ± 12 mg kg^−1^. The annual mean, maximum, and minimum temperatures were 12.2, 34.1, and − 13 °C, respectively, while the annual average precipitation and total sunshine were 456 mm year^−1^ and 2283 h per year, respectively, in this region of the continental climate.

Three different methods of weed control in giant reed plantations were applied: (i) intercropping using sweet clover (*Melilotus officinalis* L.); (ii) application of a herbicide Glialka Star (Active ingredient: glyphosate; manufacturer: Kwizda Agro Hungary Kft., Budapest, Hungary), that was applied one time in the early spring at a rate of 2% (715 L ha^−1^) using Hand-held Garden Sprayer Pressurized Pump (Knapsack Hand Operated Pressure Sprayer; Taizhou Sunny Agricultural Machinery Co., Ltd., Zhejiang, China); and (iii) hoeing that was carried out 3 times on the 3rd April, 16th June, and 29th July. Seeds of sweet clover were kindly provided by the Department of Applied Plant Biology, University of Debrecen, Debrecen, Hungary. Authors confirm that that all experiments were performed in accordance with relevant National guidelines and regulations.

### Soil sampling

To assess the impact of these three weed control methods on soil health, soil samples were collected 25 cm away from the main culm of giant reed plants in the 0–20 cm layer. Soil samples were collected in two seasons (spring and autumn) in triplicate, making a total of nine sub-samples per treatment.

The soil samples were collected in polyethylene bags and kept on ice during transportation. In the laboratory, after removing plant debris and gravel, the samples were sieved with a 5-mm mesh screen and stored at − 20 °C for further analysis. For chemical measurements, air-dried soil samples were grounded using stainless steel crushing machine (Model: H-4199; Humboldt Scientific, Inc., Atlantic Ave, USA) for homogeneity and sieved through a 2-mm mesh.

### Analysis of soil respiration and enzyme activities

Emitted CO_2_ was used as a function of microbial activity in the soil. The CO_2_ production was measured after 10 days of incubation with NaOH-trapping at 35 °C^46^. However, before measuring the soil respiration and the activity of soil enzymes the frozen soil samples were left out of the freezer for 24 h and amended with glucose at a rate of 80 mg g^−1^ after adjusting its moisture content to 60% of the total water capacity to induce microbial growth after the dormancy period. Dehydrogenase activity in soil was determined using the reduction of 2,3,5-triphenyl tetrazolium chloride (3% w/v TTC) method^47^. The absorbance of the solution was measured at 485 nm. The standard curve was made using triphenyl formazan (TPF). Phosphatase activity was estimated following the protocol described by Dick et al.^48^. The soil sample was incubated with p-nitrophenyl phosphate; the generated color intensity was measured colorimetrically at 440 nm. Urease activity was measured by the method of Kandeler and Gerber^47^ after incubating the soil sample with a 2% aqueous urea solution, and the residual urea was determined colorimetrically at 527 nm. Catalase activity was determined following the protocol described by Guwy et al.^49^. A spectrophotometer (Amersham Biosciences Ultrospec 2100 Pro UV/Visible; Holliston, MA, USA) was used to accomplish all the previous measurements.

### Determination of physicochemical properties of soil samples

The saturation percentage (%) of the soil was determined using the method described by Sparks et al.^50^. Soil pH was measured in 1:2.5 (w/w) soil: distilled water suspension^50^. Soil electrical conductivity (EC_e_), dS m^−1^, was determined in soil paste extract at 25°^50^. Soil organic matter (SOM) content was determined by the Walkley–Black chromic acid wet oxidation method^50^.

The analysis of soil total N content and its speciation (NO_3_^−^-N, NH_4_^+^-N, and Org-N) were measured using the Kjeldahl method^50^. Extractable P was extracted according to Olsen et al.^51^ using 0.5 N sodium bicarbonate and determined spectrophotometrically (Amersham Biosciences Ultrospec 2100 Pro UV/Visible; Holliston, MA, USA) by ascorbic acid method^52^. Available potassium was extracted with the ammonium acetate extraction method described by Sparks et al.^50^ and determined using a flame photometer (Sherwood Model 410; Incheon, South Korea) instrument according to the method described by Jackson^53^.

### Statistical analysis

Dependent variables were checked for normality and homoscedasticity and transformed as necessary. Data analysis was performed using Microsoft Excel 2003 (mean values ± standard deviation) and the SPSS 13.0 software package (SPSS Inc., Chicago, IL). The analysis of variance using two-way ANOVA was conducted on the interaction between treatments and sampling time, while one-way ANOVA was applied to evaluate the differences among treatments within one sampling time and within the two sampling times. Separation of means was performed by post hoc test (Tukey’s test), and significant differences were accepted at the levels *p* ≤ 0.05, 0.01, and 0.001.

## Conclusion

Weed control in giant reed plantations using the sweet clover intercropping technique resulted in higher activities of soil enzymes. On the other hand, glyphosate herbicide and hoeing negatively affected soil chemical and biochemical characteristics. Thus, controlling weeds in the giant reed plantations by intercropping, especially with sweet clover, leads to significant improvements in soil properties. These improvements in soil properties enhance the growth of giant reeds. To our knowledge, this paper is the first report that displays the effect of different weed control methods in biomass plantations and their impacts on soil's physical, chemical, and biochemical properties.

## Data Availability

All data are available within the text.
